# Controlling a new plasma regime

**DOI:** 10.1098/rsta.2023.0403

**Published:** 2024-08-26

**Authors:** M. Lennholm, S. Aleiferis, S. Bakes, O. P. Bardsley, M. van Berkel, F. J. Casson, F. Chaudry, N. J. Conway, T. C. Hender, S. S. Henderson, B. Kool, M. Lafferty, H. F. Meyer, J. Mitchell, A. Mitra, R. Osawa, R. Otin, A. Parrot, T. Thompson, G. Xia, the STEP Team

**Affiliations:** ^1^ United United Kingdom Atomic Energy Authority, Culham Campus, Abingdon, Oxon OX14 3DB, UK; ^2^ DIFFER—Dutch Institute for Fundamental Energy Research, De Zaale 20, Eindhoven 5612, Netherlands

**Keywords:** plasma control, tokamak, nuclear fusion, MIMO control

## Abstract

Success of the UK’s Spherical Tokamak for Energy Production (STEP) programme requires a robust plasma control system. This system has to guide the plasma from initiation to the burning phase, maintain it there, produce the desired fusion power for the desired duration and then terminate the plasma safely. This has to be done in a challenging environment with limited sensors and without overloading plasma-facing components. The plasma parameters and the operational regime in the STEP prototype will be very different from tokamaks, which are presently in operation. During fusion burn, the plasma regime in STEP will be self-organizing, adding further complications to the plasma control system design. This article describes the work to date on the design of individual controllers for plasma shape and position, magneto hydrodynamic instabilities, heat load and fusion power. Having studied ‘normal’ operation, the article discusses the philosophy of how the system will handle exceptions, when things do not go exactly as planned.

This article is part of the theme issue ‘Delivering Fusion Energy – The Spherical Tokamak for Energy Production (STEP)’.

## 1. Introduction

Research into the generation of electricity from nuclear fusion is normally referred to as ‘Controlled Thermonuclear Fusion’. As such, ‘control’ is in the name, highlighting how central ‘control engineering’ is to ensure that a fusion reactor can achieve its goal. Consequently, control aspects have been involved in guiding the design of the Spherical Tokamak for Energy Production (STEP) prototype power plant from an early stage [1–5]. Like the ITER [6] and the European demonstration power plant - DEMO [7] projects, the STEP prototype will be a tokamak, characterized by a high toroidal magnetic field and a high toroidal plasma current. This means that many of the challenges encountered are similar to those discussed in [6,7]. Similar efforts are ongoing to design the plasma control systems for DEMO [8] and ITER [9], with the ITER design being significantly more mature than the STEP design presented below. The main difference with respect to ITER and DEMO is that the STEP design is based on the ‘spherical’ tokamak principle as discussed below.

**Figure 1.**
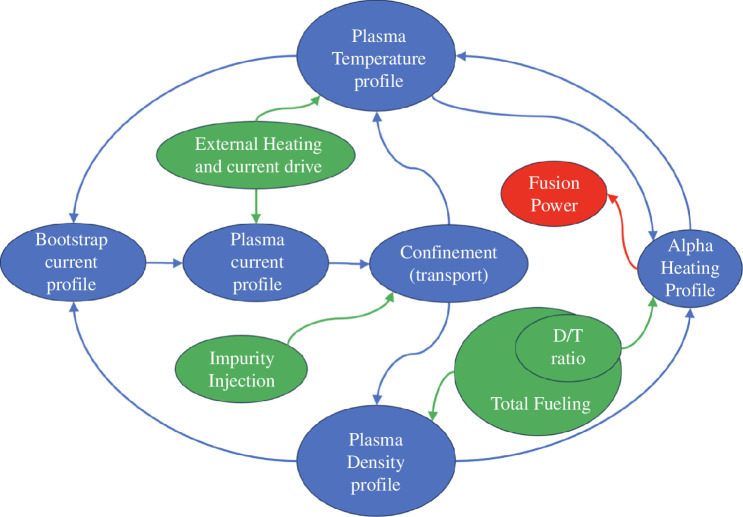
Self-organizing system. The green-shaded items represent the main actuators in the system. Actuators to control plasma stability, plasma shape and position and a number of other quantities are also required; for simplicity, they are not included in this figure, although their action is likely to affect the self-organizing system through their influence on the plasma confinement. Note that the deuterium/tritium (D/T) ratio will not be available for burn control during the main steady-state phase as most fuel will be recycled without separating D and T.

**Figure 2.**
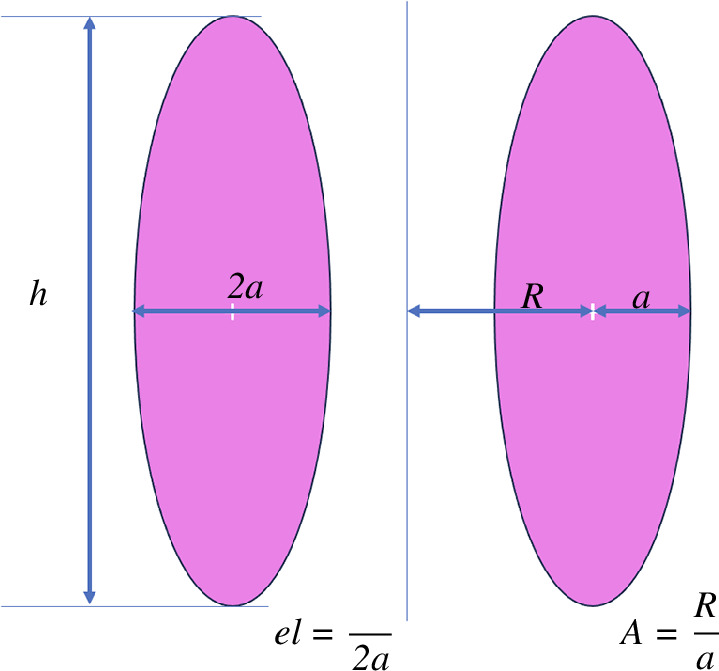
Definition of the main plasma dimensional parameters: minor radius (a), major radius (r), aspect ratio (A) and elongation (el).

When moving from present tokamaks to power-producing reactors new fundamental control challenges are encountered. Like the ITER and DEMO systems, the STEP plasma control system has to control a burning plasma, where the alpha particles generated by the fusion reaction will constitute the main plasma heating source, reducing the need for external heating. Minimizing the external heating requirement is highly desirable, but it means that less power is available for controlling the plasma. In contrast to ITER and DEMO, where the plasma current will be driven by external means, the plasma current in the STEP prototype will be dominated by the so-called ‘bootstrap’ current [10]. This current is ‘auto-generated’ by the plasma and STEP will therefore enter into a new plasma regime where both plasma current drive and heating are mainly generated by the plasma itself. The amplitude and radial distribution of the bootstrap current depend on the local gradient of the plasma pressure. As a consequence, the plasma current and its radial profile are mainly determined by the plasma temperature and density radial profiles, which in turn are governed by the alpha particle radial distribution. The alpha-particle distribution is itself determined by the density and temperature profiles. Furthermore, the plasma current profile influences the plasma confinement and, through this, both the temperature and density profiles. In the burning state, the plasma therefore constitutes a highly self-organized system. It is the task of the plasma burn controller to break into this system to ensure that the plasma reaches the state enabling it to produce significant fusion power. Once in this state, the burn controller must control fusion power to the desired value. Figure 1 illustrates the interdependence of the different aspects of this self-organized system. Operating with a high bootstrap current fraction requires operation at high normalized plasma pressure $\beta_{\text{N}}=2\mu_{0}\frac{\langle p\rangle a}{B_{\text{t}}I_{\text{p}}}$ , where a is the minor radius, $B_{\text{t}}$ is the toroidal magnetic field, $I_{\text{p}}$ is the plasma current and $\langle p\rangle$ is the volume-averaged pressure. Spherical tokamaks, i.e. tokamaks with a low aspect ratio $A=\frac{R}{a}$ , where *R* is the major radius of the plasma, facilitate operation at high $\beta_{\text{N}}$ and high bootstrap current fraction [11] thereby opening the way to steady-state operation with only a moderate amount of current being driven by the external heating and current drive systems. The definitions of the main plasma dimensional parameters are illustrated in figure 2.

While enabling steady-state operation, operating at high normalized pressure comes with additional control challenges. Achieving high $\beta_{\text{N}}$ and a high bootstrap current fraction requires operation at high plasma elongation $el=\frac{h}{2a}$ , where *h* is the plasma height as defined in figure 2. The vertical position of highly elongated plasmas is unstable with the instability growth rate increasing with elongation, and active stabilization is required to maintain such plasma configurations. One of the benefits of spherical tokamaks is their high natural elongation [12], meaning a higher overall elongation is controllable than in conventional tokamaks. Stabilizing highly elongated plasmas does, however, constitute a significant control challenge. In addition to this fundamental instability, a second instability, the so-called resistive wall mode (RWM), is encountered when $\beta_{\text{N}}$ is raised sufficiently [5]. In this case, active RWM control will also be required. The control requirements for stabilizing RWMs are similar to those for stabilizing the plasma vertical position.

**Figure 3.**
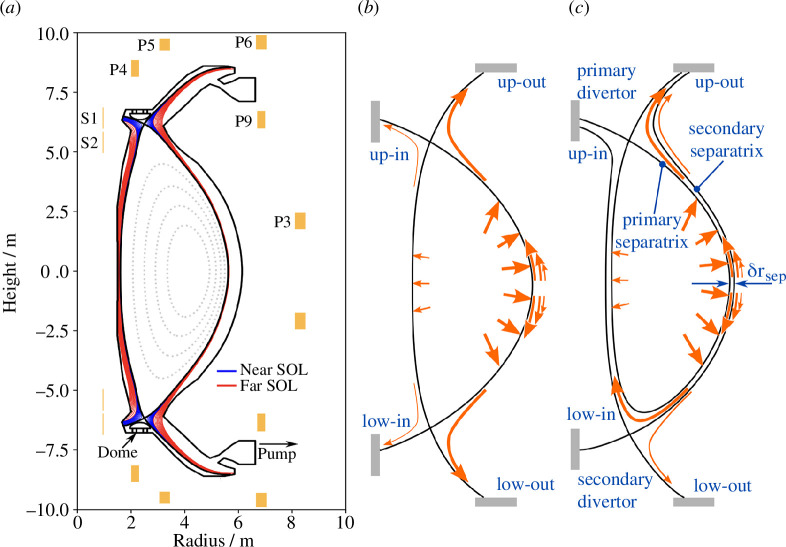
(*a*) Coil positions and plasma shape. SOL refers to the scrape-off layer, i.e. the layer of plasma just outside the confined plasma boundary, where density and temperature decay exponentially. Near SOL and far SOL somewhat arbitrarily refer to how deep into this SOL a particular part of the plasma is. (*b*) Perfect and (*c*) imperfect double null.

Eighty per cent of the energy produced by the deuterium–tritium fusion reaction escapes the plasma through 14 MeV neutrons, which are absorbed in the tritium breeding blankets surrounding the plasma [13,14]. The remaining 20% of the fusion power plus any additional power injected into the plasma, will leave the plasma and impinge on the plasma-facing components (PFCs) [15]. In a power plant, this power is significantly larger than in present-day tokamaks. To avoid damaging the PFCs this power has to be distributed over as large an area as possible. Achieving a tolerable power distribution over the PFCs requires precise control of the plasma shape, directing the charged particles leaving the plasma towards the machine ‘divertors’. These divertors are the areas where most of the charged particles leaving the plasma core impinge on the PFCs. STEP has chosen to design the machine to operate in a ‘double null’ configuration with two divertors, one at the top and one at the bottom of the machine [15–21]. Having two divertors allows the power to each of them to be approximately halved. Each of these two divertors has two ‘legs’ as illustrated in figure 3 with the outer legs shaped in a so-called ‘Super-X configuration’ to maximize the radii of the outer ‘strike points’ where the charged particles reach the material surfaces. To take full advantage of the double-null configuration, the unstable plasma vertical position has to be sufficiently precisely controlled. Although the double null design optimizes the power distribution over the divertor areas, the annular regions where charged particles impact the divertors are very narrow and the power densities will still be excessive without further measures. For this reason, most of the power needs to be radiated from the plasma such that only a small fraction reaches the PFCs in the form of charged particles. The required radiation will be induced by injecting light impurities such as argon and xenon into both the main plasma and the plasma in the divertor regions. As the radiation from the plasma is essentially isotropic, radiating most of the power allows it to be distributed over a much larger area. Injecting impurities—i.e. non-fuel particles—to induce radiation comes at the cost of reducing the plasma performance—i.e. the fusion power—and therefore good control of the impurity concentrations is required to strike the best compromise between PFC heat loads and fusion power performance. In addition, the appropriate flux of impurity and hydrogenic particles should be injected into the divertors to induce the power, momentum and particle losses required to achieve acceptable divertor target conditions.

The burning plasma environment not only challenges the PFCs, it also makes the implementation of actuators and measurements much more difficult than in current devices. The main issue that affects actuators and especially sensors is the intense neutron radiation. These neutrons will be slowed and absorbed in the blanket and shielding surrounding the plasma, but a significant amount of neutron radiation will still impinge on actuator and measurement equipment. While the actuators foreseen to be used in STEP are well defined [5,13,22,23], developing sufficient measurement capability to allow the required control performance remains a significant challenge and the controllers have to be adapted to operation with alternative and limited measurement capabilities.

Controlling the plasma during normal operation only constitutes a fraction of the tasks for which this system is responsible. The system also has to respond correctly to the occurrence of faults or undesirable plasma behaviour, the occurrence of which can have a significant impact on the plant and its operations due to the potential for loss of control of the plasma. Such loss of control may lead to an uncontrolled plasma termination (a disruption), which can have a range of consequences [24,25]. As a minimum, availability of the plant will be reduced owing to the need to re-condition the PFCs, followed by the re-energization of confinement magnets. More serious disruptions are likely to cause damage to tokamak components resulting from the uncontrolled release of thermal and magnetic energy contained within the plasma. Where this requires replacement of in-vessel components, significant operational time will be lost [26]. In the worst case, the unmitigated release of energy by a disruption in a fusion power plant has the potential to cause catastrophic damage to the tokamak machine.

For the reasons above, plant faults or abnormal plasma behaviour, collectively referred to as ‘exceptions’, need to be identified and mitigations put in place to prevent or limit their impact. Exception-handling for plasma disruption avoidance or mitigation is primarily focused on the protection of the asset rather than for the provision of safety, since only limited safety claims are expected to be based on the plasma control systems owing to their complexity and the inherent difficulty in qualification of such systems. Safety claims shall primarily be placed on the design of the machine and its containment structures.

Exceptions can occur as a result of plant failures resulting from poor design, maintenance or operations as well as from poor security and mal-intent. Exceptions can also arise in the plasma itself through perturbations in the plasma density, pressure and kinetic profiles arising as a result of complex physics phenomena. Already in the design phase, efforts must be made to eliminate or mitigate potential exceptions ‘by design’. Including an appropriate amount of redundancy is essential to reduce the impact of loss of actuator or sensor capability. To back this up, automated protection functions shall be implemented, responding to escalating exceptions, detecting the onset of exceptions and initiating the most appropriate response. Such systems will typically need to act in a few seconds with some events requiring responses within a second or less. Such fast response will be significantly more difficult to achieve than in present tokamaks as a result of the self-organizing character of the burning plasma. While it is possible to simply ‘switch off’ the plasma, this itself would lead to a disruption and therefore appropriate ‘termination scenarios’ need to be implemented, which can terminate the plasma safely within the time available.

The exception-handling needs for a fusion machine require complex condition-dependent decision-making within seconds of off-normal events being detected and with severe consequences of failure. To this is added the difficulty of monitoring such events within the harsh tokamak environment.

The remainder of this article is organized as follows: §2 describes the various individual controllers required for STEP operations in more detail. §3 discusses ‘exception handling’ while §4 summarizes measurement and actuator considerations. Finally, §5 draws some conclusions.

## 2. Controllers

**Figure 4.**
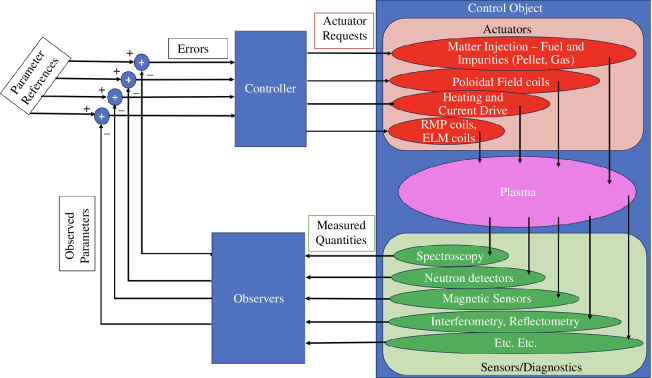
Overall plasma control loop, showing actuators, sensors, observers and controllers. The list of actuators and in particular sensors is far from exhaustive.

A wide range of tasks have to be achieved by the STEP plasma control system. Figure 4 shows a traditional view of this integrated control system where a range of parameters have to follow certain references. To achieve this the control system needs to have information about the actual parameter values to compare with the desired values. These parameter values are likely not to be directly measurable and hence they need to be derived through more or less complex algorithms based on the measurable parameters. The algorithms used to derive these parameter values are normally referred to as ‘observers’ as illustrated in the figure. Figure 4 shows the system as a large multi-input multi-output system. It is desirable to split this system into a number of separate controllers controlling only a few parameters each. The main individual plasma control tasks are discussed in the following.

### (a) Plasma position and shape control

The shape and position of the plasma are controlled by varying currents in a mixture of superconducting and resistive poloidal field coils situated around the plasma as shown in figure 3*a* [27]. The ability to vary the current in the superconducting coils is limited due to alternating current (AC) losses, which, if excessive, will lead to the coil heating up and eventually losing its superconductive properties resulting in a ‘quench’. Such quenches have to be avoided as they may severely damage the coil and hence only slow current variations can be tolerated for these coils. Faster control will therefore need to rely on the resistive coils. To reduce resistive losses and keep coil temperatures within operating limits, it is desirable to maintain the direct current (DC) in resistive coils at or near zero and hence the plasma position and shape controller should ensure that low frequency and DC current is generated in the superconducting coils while AC is generated in the resistive coils.

Conducting structures between the plasma and the control coils will slow down the penetration of the magnetic fields generated by the coils through currents induced in these structures. Similarly, variation in the magnetic fields generated by the plasma and observed by magnetic sensors, will be temporally ‘filtered’ by any conducting structures between the plasma and the sensors. This filtering of both the control fields and the observed field will limit the control bandwidths of the plasma position and shape control system. While such conducting structures hamper fast control action, they also damp any fast variation of the plasma position and shape itself because currents induced in these structures counteract any plasma movement. For this reason, the conductivity distribution of elements situated near the plasma has to be selected carefully to strike the best compromise between stabilizing the plasma and allowing sufficiently fast active control.

Getting this compromise right is particularly important to stabilize the unstable vertical plasma position. Any elongated plasma is vertically unstable and requires active stabilization. The growth rate of this vertical instability would be too large to be controlled in the absence of stabilizing induced currents in conducting structures surrounding the plasma. The current induced in toroidally conducting structures will create magnetic fields counteracting the vertical movement. This induced current will be up-down antisymmetric with respect to the machine midplane. This allows any toroidally conducting loops to be broken at one toroidal location and connected in anti-series. Although anti-series connection is not required to achieve the stabilizing effect, this connection prevents up-down symmetric currents from flowing in these structures. This is important to ensure that current due to flux from the central solenoid at the time of plasma formation is induced in the initial plasma rather than in the conducting structures.

While induced stabilizing currents will reduce the growth rate to manageable levels, controlling the vertical instability will still require a fast active control system. For the present design, growth rates of approximately 10 s^−1^ are expected during the plasma ramp up reducing to approximately 1 s^−1^ during the flat top. Such growth rates are low in comparison with present tokamaks. This is due to the size of the STEP prototype with the associated slow inductive decay times. These modest growth rates do not, however, reduce the control challenge due to the required large stabilizing power associated with the large plasma current. An active stabilization system will drive up/down antisymmetric currents in resistive coils situated above and below the midplane outside the breeding blanket using a powerful fast amplifier. This current will be controlled to respond to measured variation of the plasma vertical position and velocity. If this control system fails, the plasma will undergo a vertical displacement event (VDE). It will accelerate either up or down, eventually contacting the PFCs and shrinking until it disrupts. In such VDEs all the energy of the plasma is lost to the surrounding components and very large currents are generated in these components. These currents result in large forces being produced as a result of local electric currents flowing perpendicular to local magnetic fields (J × B forces), risking significant damage to the machine. Robust vertical stabilization is therefore essential for STEP.

The maximum achievable plasma pressure increases with plasma elongation, so it is desirable to increase the elongation as much as possible, while maintaining tolerable vertical instability growth rates. For constant elongation, the growth rate decreases with increasing induced toroidal currents in the conducting structures. Furthermore, the radial distribution (profile) of the plasma current affects how much current is induced in the conducting structures. A broader plasma current profile (lower internal inductance $l_{i}$ ) has more current near the stabilizing structures and hence sees a stronger stabilizing effect of these structures. Here, $l_{i}=\frac{\int_{,}^{V}B_{p}^{2}dv}{{\left(\frac{1}{{R_{2}-R}_{1}}\int_{-R_{1}}^{R_{2}}B_{p}\left(r\right)dr\right)}^{2}}$ , where $B_{p}$ is the poloidal magnetic field, *V* is the plasma volume, *v* is the volume element and $R_{1}$ and $R_{2}$ are the major radius of the inboard and outboard plasma boundary at the plasma midplane. Thus, good control of the plasma current profile is required to enable operation at high elongation and high normalized pressure.

As described in §1, the selected double-null design effectively doubles the surface area over which the exhaust power is distributed. It furthermore isolates the inner targets—which have the smallest surface area—from the outboard scrape-off layer (SOL), which will receive most of the power (see figure 3*b*). To take full advantage of this, the vertical position must be controlled to a degree of accuracy not much greater than the heat flux decay length, which is of the order of millimetres, in order to minimize the amount of power flowing between the separatrices (see figure 3*c*).

Assuring satisfactory real-time determination of the vertical position and vertical velocity is one of the fundamental measurement challenges that must be solved. Substantial efforts are being put into investigating a range of options and assessing their reliability in view of the harsh environment that will be encountered during high power operation. In current tokamaks, the vertical motion of the plasma is determined using magnetic measurement coils situated around the plasma. In STEP, such coils will have to be situated behind the blankets, and the conductivity in elements between plasma and pick-up coils will filter the magnetic field penetration between the plasma and such coils in a similar way that the control fields from the active coils are filtered. This is another effect that must be taken into account when determining the conductivity distribution. While it is likely that magnetic measurement coils situated behind the blankets will be sufficiently shielded from neutrons to remain an option for STEP, the measured voltage $V_{c}$ across such coils is proportional to the time derivative of the local magnetic field $V_{c}=k\frac{dB}{dt}$ , where *k* is a calibration constant. To find the absolute value of the magnetic field B, which is required to find the plasma position, these signals need to be integrated over time: $B=\int_{0}^{t}V_{c}\left(t\prime \right)dt\prime$ . For steady-state operation, such integration will invariably drift, rendering the absolute measurement invalid. Thus, magnetic coils can be used to measure the vertical velocity of the plasma but alternative—possibly slower—measurements will be required to measure the vertical plasma position. Ultimately, it is the distribution of the heat load between the upper and lower divertors that matters, so a measurement of the ratio of the heat conducted towards the two divertors may provide sufficient information. Other options that may provide information about the plasma position could be interferometers, reflectometers and anything where real-time tomography is possible.

Although the radial plasma position is not unstable, good control is still required to ensure that rapid changes in plasma conditions do not lead to excessive radial movements resulting in the plasma contacting the inner or outer first wall. If the plasma loses significant stored energy, for example, through the transition from high confinement (H-mode) to low confinement (L-mode), the plasma will move inwards by a significant distance. To avoid the plasma contacting the inner wall in this case, the plasma has to either be sufficiently far from the inner wall before the transition, or the control system has to respond sufficiently quickly to counteract this movement. From a fusion performance point of view, it is important to operate with the plasma as close as possible to the inner wall. This allows the spherical tokamak advantage to be maximized, making optimal use of the available toroidal magnetic field and reducing the overall machine size requirement. Hence, increasing the plasma wall distance is not desirable. The best solution is likely to be a compromise between a powerful and fast control system based on resistive coils and sufficient plasma-wall distance to accommodate the inevitable plasma movements.

### (b) Resistive wall mode control

The physics of the RWM in STEP is discussed in the paper ‘Plasma Burn—Mind the Gap’ in this issue [5], where it is concluded that active RWM control is prudent for STEP. The RWM is the non-axisymmetric analogue of the vertical instability (or more correctly the vertical instability is an axisymmetric RWM) [28–33]. Like the vertical instability, control of the RWM requires conductors relatively near the plasma to slow the instability growth rate to the point where active feedback control with coils is possible. Various RWM control studies for STEP have been conducted using the MARS-F code [34], some of which are detailed in [35]. RWMs are stable even without any stabilizing conducting structures when the plasma pressure is less than a certain threshold. Above this threshold active stabilization is required and such stabilization becomes increasingly difficult as the pressure increases. The plasma pressure in the STEP prototype is expected to be marginally above this threshold.

**Figure 5.**
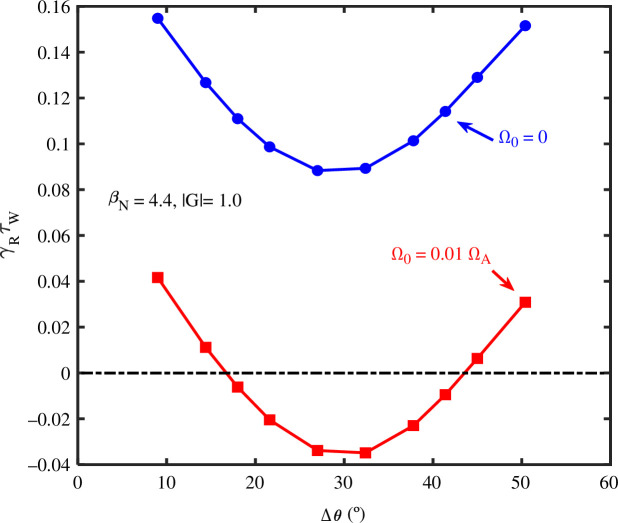
Calculations of the feedback-controlled growth rate $\;\gamma_{R}\tau_{W}$ (normalized to the wall time) versus the poloidal angular extent of the coil. Results are shown for two parabolic toroidal rotation profiles with central values $\Omega_{0}=0.01\Omega_{A}$ and ${\text{Ω}}_{0}=0$ ( $\Omega_{A}\;is$ the Alfven frequency).

Modelling for the *n* = 1 RWM shows that for the pressures expected, ex-vessel RWM control coils are likely to be an option, but they require substantially more power than internal coils. At higher pressures, they fail to give stabilization. It is thus concluded that the RWM stability should be provided by in-vessel coils. The RWM control coils are planned to be located just behind the blanket, to provide shielding from the fusion neutrons and will be copper alloy coils, probably relatively similar to the ITER edge localized mode (ELM) coil design [36]. As it is desired to be able to stabilize both *n* = 1 and 2 RWMs, eight regularly spaced coils in the toroidal direction are planned. Midplane and pairs of coils above and below the midplane were examined as options for RWM control. It was found that midplane coils were more effective and further studies concluded that the optimal poloidal angular extent of the midplane coils is approximately ±15° (see figure 5). As detailed in [35] a feedback control scheme with a simple proportional differential controller, and white noise with a given standard deviation applied sensor signal, has been studied. The sensor, which measures the *n* = 1 poloidal magnetic field, is assumed to be behind the first wall at the same radius as the control coils. This means that measurement phase delays due to the first wall eddy currents are included in the simulations. For these feedback calculations with MARS-F, the primary passive stabilization of the RWM is assumed to be due to a continuous first wall; in practice, it is likely the RWM passive stabilization will be provided by currents in the blanket modules or copper loops near the plasma. Three-dimensional electromagnetic modelling is in progress to design this passive system. It is likely that the derivative feedback term will require a magnetic sensor measurement as input, but the mode amplitude might be measured by a slower response system such as microwave reflectometry or electron cyclotron emission [37].

### (c) Burn control and control of the self-organizing plasma profile system

Controlling the fusion power produced in STEP is one of the more obvious tasks of the plasma control system. When looking into such control in more detail, it becomes clear that this task is intricately linked to the control of the total plasma current as well as the plasma’s current, temperature and density profiles. The most demanding part of controlling this system is probably associated with ensuring that the desirable start-up trajectory is followed between plasma initiation and full-fusion burn and during the plasma termination phase. In the plasma start-up phase, all plasma heating and current drive will come through external means. The control systems have to ensure that the transition from this phase to the burn phase, where the plasma heating is dominated by fusion-produced alpha particles, is safely achieved [38]. Similarly, the system has to ensure that the fusion power is extinguished and the plasma current is ramped down in a controlled manner. Once significant fusion power is produced, the controller will only be able to indirectly control the plasma temperature and current profile as discussed in the Introduction and illustrated in figure 1. The control system can break into this loop by using the matter (fuel and impurity) injection systems and the heating and current drive systems. This gives a measure of control over temperature and density profiles. Optimization of the ramp up to burn has been initially done using the RAPTOR (RApid Plasma Transport Simulator) code, which has trajectory optimization capabilities [39–42]. The results that have been found have subsequently been verified using the higher-fidelity JINTRAC suite of codes [43,44]. RAPTOR, which is a control-oriented code, has furthermore been used to investigate the ability to actively control the fusion power. These control simulations indicate that the plasma can be controlled as desired, though the development of the measurement systems required by the controller to ensure that the plasma profiles follow the desired trajectories remains a serious challenge.

### (d) Heat load control

#### (i) Detachment control

The total power heating the plasma in STEP will be the sum of the fusion-produced alpha-particle heating and the externally applied heating and current drive power. All this power will impinge on the PFCs [15].

**Figure 6.**
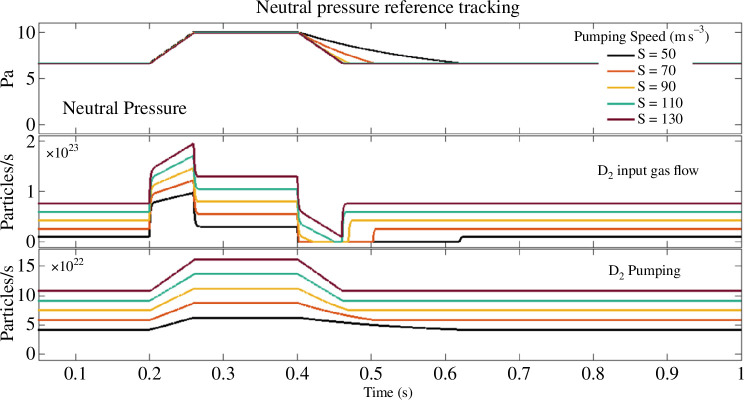
The effect of pumping speed on the ability to control neutral pressure in the divertor.

Even with the heat load reduction achieved in the double-null configuration, the power densities at the strike points will remain above acceptable limits and additional measures are required. Distributing the power over larger areas can be achieved by ensuring that most of the power is radiated, as mentioned above. Injecting a combination of impurity and hydrogenic gasses into the divertor can drive the plasma into the ‘detached’ state, where the ionization region is not in contact with the divertor targets [45]. In this case, a ‘detachment front’ is established along the divertor legs, between the strike points and the poloidal field null (X-point). The position of this front depends on the amount of power and the rate of particles being conducted from the main plasma towards the divertor and on the amount of impurity and fuelling gas being injected into the divertor region. Good control of the detachment front position is required to ensure that it neither moves to the X-point nor to the strike-point when the plasma conditions vary. To retain tight control of the detachment front position, it is essential not only to be able to inject gas (impurities and fuel) sufficiently fast, but also to be able to remove this gas quickly enough. This determines the required divertor vacuum pumping speed. Figure 6 shows a simulation of the dynamic ability to vary the vacuum pressure in the divertor as a function of the pumping speed. This acts as a good proxy for the influence of the vacuum pumping on the achievable detachment-front controller response time, as the resulting detachment state is a direct consequence of the neutral pressure [46]. To achieve robust detachment control, sensing technologies that can give sufficient real-time information are required. A number of viable options are being considered, of which the system described in [47] is an example. Whether such a system can be made compatible with the high-power phase remains to be seen. Further details of the sensing challenge are beyond the scope of this article.

**Figure 7.**
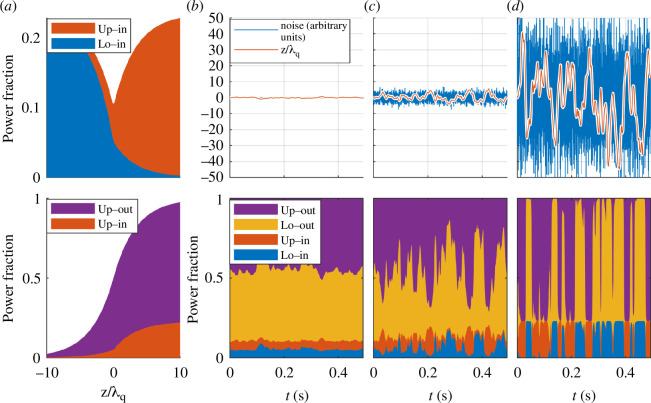
Power distribution between divertor legs as function vertical offset from ideal double null. (*a*) The fraction of the power conducted towards the divertors that will: (top) go towards the inner strike points, (bottom) go towards the top strike points. (*b–d*) The fraction of the power that will go towards each of the four strike points for three different noise levels. Top: Simulated measurement of induced current in passive stabilizers (blue). This translates, through the closed loop control loop, into vertical bouncing (orange). Bottom: Power distribution between the four strike points as a function of time.

Various disturbances will affect the detachment-front position. Preliminary simulations using the JINTRAC code have been used to assess the effect of midplane fuelling pellet injection on the detachment-front position. These simulations seem to indicate that the controller does not have to respond to individual pellets, which means that response to variations in pellet fuelling rate can be performed on a slower timescale. A more concerning disturbance is associated with double-null operation. Given the vertical instability, the plasma will always ‘bounce’ up and down around the required vertical position. If this bouncing can be kept sufficiently small the power variation on the divertor legs will remain small, but if the bouncing increases sufficiently the plasma will eventually bounce between directing all the power to the upper and lower divertors. Figure 7 shows a sketch of how the power to the four divertor legs might vary for different bouncing amplitudes. This sketch is based on very simplified assumptions regarding the power leaving the plasma towards the inboard and outboard scrape-off layer, respectively. Based on these simplified assumptions, the fraction of the power going to the different divertor legs depends mainly on $\frac{Z}{\lambda }$ , where *Z* is the vertical deviation from perfect double null and (λ) is the scale length of the exponential plasma density decay in the scrape of layer. In figure 7*a*, the top trace shows the total power going towards the inner divertor legs and the bottom trace shows the total power going to the upper divertor legs as a function of $\frac{Z}{\lambda }$ . The most notable observation to be made from this is the significant reduction of power towards the inner strike points, which can be achieved with good control of the vertical position. This information is used to generate the power distributions in figure 7*b*–*d*. To generate these figures, three different levels of the noise on the vertical position measurement have been assumed and a simple proportional–derivative (PD) controller has been implemented to control the simulated plasma position in response to the measured vertical position. This figure illustrates that increasing measurement noise will result in increasing bouncing of the vertical position. Clearly, the amplitude and frequency of such bouncing will depend on details of the design of the vertical position control system, but the importance of reducing the measurement noise as much as possible is evident. If significant bouncing cannot be avoided, the detachment controller will have to respond appropriately. It is unlikely that the detachment controller can respond quickly enough. Therefore, it is likely that the vertical position controller has to ensure that the bouncing is deterministic, so that the detachment controller can anticipate which power variation to expect. In this case, more advanced and faster matter injection systems will also be needed to achieve the required prompt variations in particle flux.

#### (ii) Core radiation control

While good detachment control would distribute the exhaust power over a larger divertor region, it is unlikely that the full exhaust can be handled by the divertors and hence the design anticipates a significant fraction of the power to be radiated from the core plasma. Ensuring sufficient core radiation allows the exhaust power to be distributed over the entirety of the PFCs, reducing the peak heat load significantly. The required core radiation is a compromise between several considerations. For the plasma to remain in high confinement mode (H-mode), the power conducted across the plasma boundary has to be sufficient to stay above the so-called H-mode threshold. This limits how much-radiated power can be tolerated. On the other hand, all the power not radiated from the core will reach the divertors, so this power has to be sufficiently small to allow effective detachment control. The core radiation will be controlled through the injection of impurities into the core. It is expected that these impurities will be incorporated into the fuelling pellets. It is desirable that these impurities cause radiation close to the plasma boundary, but it is highly likely that some impurity particles will make their way into more central parts of the plasma, reducing the fusion power generated by the plasma. Thus, the core radiation control has to ensure that the power conducted across the plasma boundary is (i) small enough for the detachment controller to cope with, and (ii) large enough to stay in H-mode. In addition, the controller should minimize the radiated power while fulfilling (i).

#### (iii) ELM control

In current tokamaks the high confinement (H-mode) regime is normally associated with repetitive sizeable, short bursts of particles and energy expelled from the plasma and impacting the divertor surfaces [48,49]. Extrapolation of these ELMs from current machines to STEP indicates that the instantaneous power reaching the divertors during an ELM is so large that it cannot be sufficiently mitigated by the detachment control and that the energy will impact the divertor surfaces in very narrow annular regions around the strike-point locations. According to present estimates, unmitigated ELMs would cause significant damage to the divertor and hence, it is unlikely that such ELMs can be tolerated. Although reduction of the ELM size may improve the situation it is doubtful if even smaller ELMs are acceptable in steady-state operation. It is therefore proposed to operate STEP in a regime either with a different type of more benign ELMs or without ELMs altogether [50]. H-mode operation without ELMs has been demonstrated under specific conditions in various tokamaks, but it is not guaranteed that such operation can be replicated on STEP. As a consequence, STEP proposes the installation of resonant magnetic perturbation (RMP) coils, which can introduce a steady, toroidally asymmetric field that has been seen to reliably eliminate ELMs, although this has not been demonstrated in spherical tokamaks [51,52]. As RMP coils reduce the fusion performance, it is desirable to use them only when needed and, for this reason, controllers are required that detect the onset of ELM activity and activate the RMP coils to eliminate the ELMs. Such control has been shown on K-STAR [53]. This scheme is only feasible if short bursts of ELMs can be tolerated intermittently. In any case, it is likely that some level of continuous RMP field will be required. Such a DC or slowly varying field will be provided by ex-vessel, superconducting RMP coils, while in-vessel resistive RMP coils will allow fast response. It should be noted that, during an ELM, the width of the scrape off layer increases significantly, such that the ELM energy is distributed evenly between upper and lower outer divertor legs even if the vertical controller is not succeeding in maintaining a perfect double-null configuration. This may mean that an operation point with some benign ELM activity can be established.

### (e) Controller interaction

Although the control issues discussed above have been treated as individual control challenges, all of the controllers interact with each other. The interaction already described of the detachment and vertical position controller is an example of such interaction. In fact, the entire plasma control system is a large multi-input multi-output (MIMO) controller. It is, however, desirable to reduce the complexity of the controller by dividing it into a number of separate single-input single-output (SISO) and MIMO controllers where each can be treated individually and where any interactions can be treated as disturbances. The tasks that the overall control system must perform will also change throughout a plasma discharge and a supervisory system will be incorporated within the plasma control system. The role of this supervisory system is to select which controllers are active and what goals these controllers need to fulfil. Changes to the goals may occur at predetermined times or they may be initiated by the occurrence of certain events. In this context, these events are considered to be within normal operation, in contrast to exception events, which are discussed in the following section. Typical ‘normal’ events would be transition from limiter to divertor operation, transitions between low (L-mode) and high (H-mode) confinement, operator intervention to change set points and many others. Other ‘normal’ events could be minor recoverable deviations from the nominal operations point. The difference between ‘normal’ events and ‘exceptions’ is defined by the assertion that the recovery of an exception may fail, leading to an escalation of the fault, whereas a ‘normal’ event is not linked to an increased risk of an exception occurring. The way to handle events and exceptions and assure optimal use of the actuators available to the plasma control system is complex and this work is still at its infancy within the STEP project. Such work has advanced much further elsewhere, for example, in the development of the SAMONE (Supervisory control and Actuator Management with Off Normal Events) supervisory controller [54] and STEP aims to take advantage of this when developing the event handling system further. The handling of exceptions is discussed in more detail in §3.

## 3. Handling plant faults and fault escalation

An exception-handling strategy has been developed for managing the risk presented by failures that can lead to loss of operations and damage to plant systems. This exception-handling strategy considers all faults associated with plant damage or safety events. However, of specific note here are those exceptions that can lead to plasma disruptions and other plasma events, which can result in damage to the tokamak and its subsystems, for the reasons discussed previously.

The purpose of the exception-handling strategy is to enable the identification of faults, which are linked to asset damage or safety events, sometimes by complex escalation routes and to limit the consequence of such events through the application of preventative or mitigating barriers within the plant design and operations. The application of multiple barriers of defence is based on the concept of ‘defence-in-depth’, which assumes no single barrier can provide complete mitigation of risk. Therefore, multiple diverse barriers are required to provide the greatest effective reduction in overall risk of failure impact. A defence-in-depth approach has been adopted by the STEP safety team and the barriers described here for exception handling will be mapped to the associated layers of defence defined within the safety case documentation.

Not all failures are equal: some failures may result in lower plant efficiency or increased operator burden, while others can lead to rapid and catastrophic damage to plant. In addition, the cost of implementing and maintaining fault-handing systems can be considerable and adds to both capital and operating costs. To manage cost and complexity, the number of barriers between fault and event, and the integrity associated with these barriers, will be graded in relation to the level of event significance and risk reduction delivered by each barrier. To support this approach, exception-handling events will be categorized in relation to the significance of associated unmitigated impact. Categories associated with increased operator burden, reduced commercial output, minor damage and major damage will be defined and the level of risk mitigation assigned to each event barrier has to be defined. The assigned category and risk mitigation enables an integrity level to be defined for each system, dictating the level of rigour in relation to design, qualification and through-life operation and maintenance activities. Some exceptions and their associated risks may not depend on a single system, but on the interaction of a range of systems. More complex event detection and exception-handling strategies will have to be defined in this case. Where risk is unrelated to nuclear safety, integrity shall be aligned to the process industry functional safety standards.

The exception-handling strategy considers mitigating measures throughout the design and operation of the plant. Barriers are applied at every stage, with priority given to elimination and reduction in occurrence over higher level functions associated with reducing consequences and monitoring impact. Each functional level will be independent from the previous level and does not rely on data from or control being handed over by the previous function. The following general areas of risk mitigation are defined within the strategy.

### (a) Design and monitoring

— *Elimination*: Where possible the design of the plant or its operations will be changed eliminating initiating faults and any potential escalation to exception events. This is the preferred approach to mitigation as it fully removes the risk without the addition of complex control and operations. An example of this is operation with a plasma current profile, which ensures that the safety factor q remains above two throughout the plasma. This eliminates deleterious Magneto Hydro Dynamic (MHD) modes associated with rational values of *q* < 2 (such as *q* = 1, *q* = 3/2, *q* = 4/3 and so on). Here, *q* is determined at each point in the plasma as the number of toroidal turns undergone by the magnetic field line going through this point before it returns to the same poloidal position. For more details of the importance and precise definition of *q*, see [5].
— *Improve resilience*: Where failures of plant or plant operations can initiate fault sequences or are part of such a sequence, redundancy and hold-points can be included to provide additional safety margin within the system. Such measures provide additional time, barriers and checkpoints allowing operator intervention and or automated recovery actions to be taken. Examples here could be the availability of spare actuator capacity and redundant sensors. Other examples could be the design of PFCs, which can sustain extra heat load for enough time to allow control systems to act to reduce such loads.
— *Monitoring and intervention*: Where possible, facilities for operator monitoring and intervention are provided. Such facilities provide operators with an understanding of operational risk and allow informed judgement as to tolerability with regards to ongoing operations and whether any remedial or preventative action need to be taken. Typical monitoring tasks could be remote inspection of PFCs to determine whether these components are sufficiently serviceable. Whether such monitoring is possible during plasma operations remains to be seen.

### (b) Automated mitigation systems

**Figure 8.**
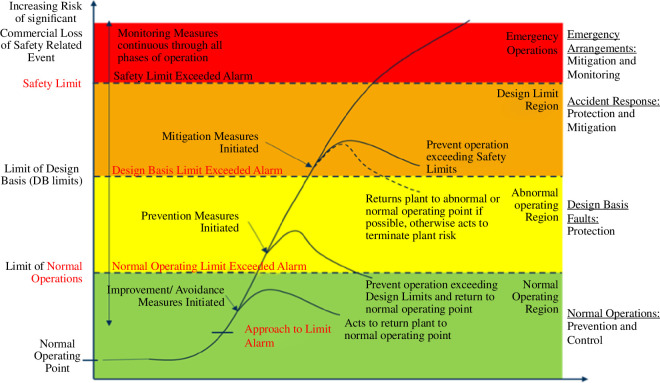
Exception-handling and plasma disruption mitigation.

Where consequences of events are significant and fast acting, it is not reasonable to expect operator intervention in preventing escalation of events. Automated systems will provide mitigation for events independent of operators through predefined event scenario management schemes (see figure 8). In order for the control system to undertake the appropriate action, real-time monitoring of the state of plasma, actuators and sensors is required and real-time algorithms will be needed to predict whether the system is moving towards a boundary in state space where action is needed. Implementing the sensors required for detecting exceptions is significantly more challenging in the STEP environment than in present machines. On the detection of an exception the system has to determine the appropriate action:

— *Avoidance*: Avoidance systems detect when the plasma state is moving away from its nominal point, while remaining within the ‘normal’ operating region and act to return the plant to its nominal state, while maintaining acceptable safety margins. Avoidance systems are generally associated with maintaining commercial operations and performance levels but in doing so provide a level of protection from exiting the safe ‘design region’ into unsafe operations. An example could be monitoring the proximity to an unintended transition from H to L mode, injecting additional heating power or reducing core radiation to stay in H-mode.
— *Prevention*: Prevention systems act to recover operations that have left the ‘normal’ operating region. These systems detect such events and initiate automated plant responses primarily focused on returning the plant to a safe condition. A ‘safe condition’ is not necessarily one that continues to provide ‘normal’ commercial operations but is one that prevents loss of future commercial performance and eliminates safety hazards. As such, prevention systems may act to shut down plant operations to preserve functional integrity for future operations. Here, an example could be the appearance of ELMs. The response could be an increase in the RMP coil currents to ensure the ELMs disappear, before potentially returning to the original operations point. Another example could be the detection of a damaging MHD mode, triggered by excessive pressure. If the mode cannot be eliminated, a slow plasma termination will be required.
— *Mitigation*: Where prevention is not possible or has not been effective, mitigation systems act to reduce the consequential impact of the failure escalation rather than to prevent them in the first place. Examples of mitigation systems include shattered pellet injection (SPI) and massive gas injection (MGI) as well as termination of plasma fuelling, where the system attempts to reduce the level of energy within a plasma before a disruption such that the impact of the disruption is as low as possible. It should be noted that mitigation systems themselves present a risk to the plant, with their initiation being likely to trigger a disruption, albeit of a more minor nature. Where possible, delays, etc, shall be provided to allow intervention by lower level functions before initiation of any higher level function such that opportunity is given to achieve a return to normal operation or safe shutdown, etc, before potentially disruptive and damaging mitigating action is taken.
— *Monitoring*: Finally, monitoring systems are provided to enable the level of damage or harm resulting from the event to be quantified. Such monitoring could involve remote camera inspection of PFCs, blanket integrity, etc. These systems are needed to meet regulator expectations and reporting requirements as well as to support commercial operations, as validation of system integrity will be required before recovery and re-start activities can be initiated. Post-event analysis can also support engineering and scientific learning and feed into improvements in the design and operation of future plants.

## 4. Actuator and measurement requirements

To achieve the desired controller performance, the various actuator and measurement requirements have to be derived from the control requirements described above. This is the reason that the plasma control system design has commenced in the early phase of the STEP conceptual design phase. Whereas the actuators that will be available in STEP are fairly well defined, the range of measurement/diagnostic systems that could be used is far wider and the definition of which diagnostics to install is still in a very early phase. This section discusses the actuators that have been chosen in §4a, describing which control tasks each actuator can contribute to. Section 4b discusses which parameters need to be made available in real time and which measurement systems could contribute to providing these parameters.

### (a) Actuators

Significant efforts have gone into selecting the STEP actuators. The main choice that has been made is for the heating and current drive system. A number of traditional systems have been considered:

— *Neutral beam injection*: Such a system would occupy a very large volume, reducing the available blanket volume. In addition, very high neutral particle energies would be required to penetrate to the centre of the plasma. For this reason, such a system has been deselected.
— *Ion cyclotron resonance heating and current drive*: To couple significant power from such a system to the plasma, radio frequency antennas would have to be located very close to the plasma and this would pose very significant engineering challenges resulting in this system being deselected.
— *Electron cyclotron heating and current drive (ECRH/ECCD)*: This system is a millimetre-wave system that can launch power into the plasma through Gaussian beams, and as such the launchers can be situated far from the plasma. This system can both heat and drive current in the plasma, and the technology is well established. As a consequence, ECRH/ECCD has been chosen as the main heating and current drive system for STEP. A fairly high toroidal field is required by this system, and this has driven the choice of the machine toroidal field in the current design.
— *Electron Bernstein wave current drive (EBW)*: This system is another millimetre-wave system, which can use the same transmission equipment as the ECRH/ECCD system. It will operate at different frequencies from the ECRH/ECCD system and it is likely to require different launchers. Very little experience exists with the use of EBW in tokamaks, but such a system has the promise of significantly increased current drive efficiency, especially for off-axis current. For this reason, STEP is planning a dual ECCD/EBW system that allows the option of using EBW if further confidence can be gained through experiments that will be undertaken on MAST-U (Mega Ampere Spherical Tokamak - Upgrade) over the coming years.

The choice of ECRH/CD/EBW for heating and current drive gives significant flexibility in controlling the location of the heating and current drive, allowing a measure of control of the current profile that is independent of the bootstrap current.

For the poloidal field coils, the main choice is between superconducting and resistive coils and a combination has been selected, with resistive coils being used for rapid control action such as control of the radial and vertical position. The aim would be to have zero DC current in these coils with any DC current requirement being carried in superconducting coils.

The main fuelling system will be based on pellet injection, as gas injection is expected to be very inefficient. The use of some gas injection is expected for impurity injection into the divertor, though the use of doped pellets may also be a good option to speed up the actuator response.

**Figure 9.**
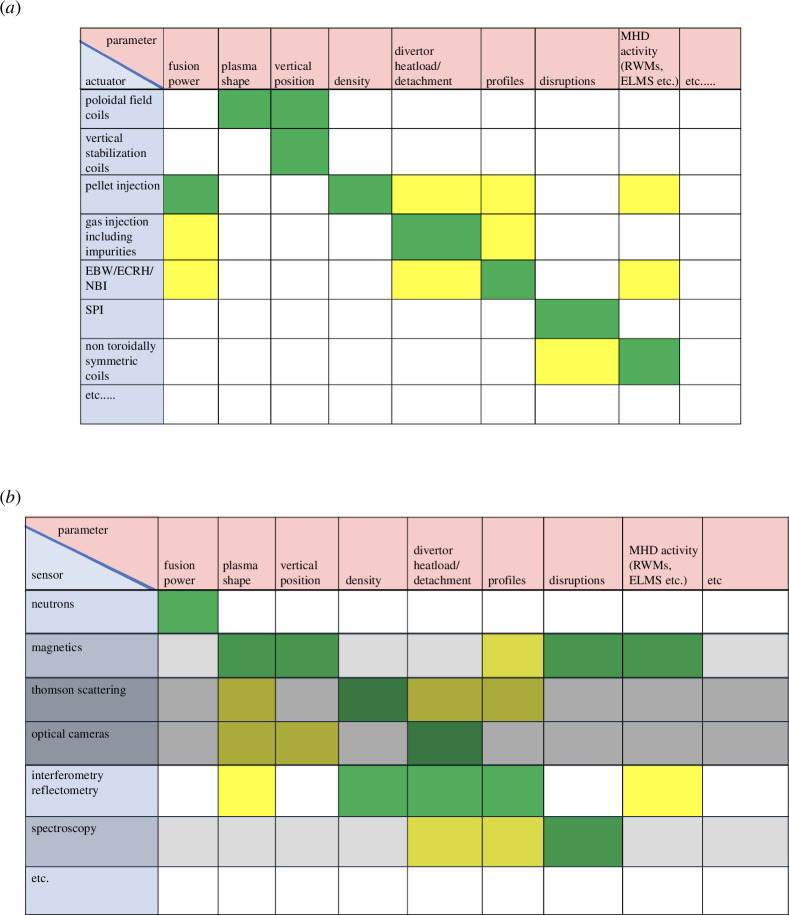
Illustrative depiction of potential (*a*) actuators and (*b*) sensors and their relationship to control tasks. The grey shading represents the likelihood of these diagnostics being compatible with full-power operation, with the darkest shading corresponding to the lowest likelihood.

The types of actuators foreseen are depicted in figure 9*a* together with the control task that they can contribute to. In general, all actuators affect all controllers, so the table is colour coded to indicate the strength of this effect, with dark green indicating that this type of actuator is likely to be used directly in the control task in question, whereas yellow indicates that these actuators have a significant influence on the control task without being the primary actuator. Neither the list of control tasks nor of types of actuators is exhaustive and each type of actuator consists of a number of individual actuators.

### (b) Parameters to measure and potential measurement systems

The various controllers described require real-time information about a number of parameters. These parameters can rarely, if ever, be measured directly. Instead, they will be derived from one or more measurements using ‘observers’. When moving to a burning plasma, the choice of measurement systems that can survive the harsh reactor environment becomes very limited by comparison with the systems used in present-day tokamaks. For example, some of the systems traditionally used will be incompatible with the high neutron rates associated with burning plasma operation. Innovative solutions are needed to get the required information from limited diagnostics. The plan for STEP is to install a wide range of diagnostics for an initial low neutron-rate phase of operations, and use these to qualify observers based on the reduced set of measurements foreseen for the full power operations phase. Figure 9*b* shows the relationship between controllers and potential measurement systems in a similar format to figure 9*a*. The shading in this figure represents the difficulty in using the sensors in question during full power operation, with darker shading representing higher difficulty. Although some measurement systems, such as optical cameras and Thomson scattering seems unlikely to be compatible with high power operation, no systems are ruled out at the moment.

**Figure 10.**
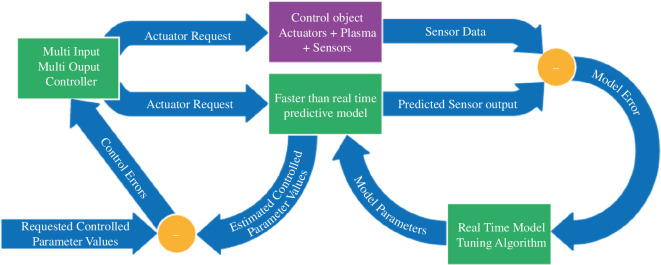
Model-based control.

To achieve robust control with limited measurement availability, the intention is to implement real-time models, which output the desired control quantities. These model quantities are then used instead of measured quantities. As such models will not match the control object perfectly, they will contain ‘synthetic diagnostics’ that produce estimates of measurable quantities, which can be compared with actual measurements. The differences between these estimates and their history and the actual measurements and their history can then be used to tune the models in real time but with less frequent updating. In this way, the outputs from the model act as an observer, allowing low-noise control to be achieved as long as the model does not need to be updated too frequently. Examples of such model-based observers and their use can be found in [55,56]. Figure 10 illustrates this principle.

Given the slowness of some of the actuators, this methodology will be taken one step further by moving to ‘model predictive control’, as demonstrated successfully on the TCV tokamak [57]. In this case the model will be running ‘faster than real time’, meaning that the model can predict the future evolution under a variety of actuator trajectories, comparing the resulting output trajectories with the desired future trajectories and choosing the best trajectory option. This process can be repeated continuously, ensuring the best possible actuation of the system. The longer the prediction horizon is with respect to the time constants involved and the more complex the real-time models, the more demanding this will be in terms of computational resources. A wide range of time constants are involved ranging from approximately 0.1–1 s for the plasma vertical instability and for RWMs through a few seconds for the energy confinement time up to several hundreds of seconds for current diffusion. These time constants govern the allowed complexity of the real-time models used for each individual control task. This process can be expanded to the response to abnormal events, where entirely new trajectories need to be found, either to recover the plasma or to terminate it safely.

## 5. Conclusions

Plasma control is central to achieving the goals of the STEP design, and hence the requirements for the design of the machine, including all actuators and measurement systems, will be informed by the plasma control design. While this work is still in its initial phase, the authors feel confident that, in the current design iteration, the plasma can be initiated, ramped up to burn and terminated with the available actuators. In addition, the way the control system will handle exceptions is being defined, with the type of response depending on the type of exception and the associated risk. Although the definition of diagnostics remains at a very early stage, a number of diagnostic options seem viable and these should be able to provide enough information to enable the required control. Much work does, however, remain to improve the confidence in the ability to gain enough real-time information about the plasma state during the burn phase. Other than improving the diagnostic capability, the confidence in the design can also be significantly increased by improving the available real-time models, with more reliable real-time models leading to reduced diagnostic requirements. Progressing the design of the plasma control system outlined in this article will require very significant work during the next phase of the STEP design phase. In this phase, the detailed controller design work should culminate in a mature design allowing machine construction to commence with confidence.

## Data Availability

This article has no additional data. To obtain further information on the data and models underlying this paper please contact PublicationsManager@ukaea.uk.
